# MicroRNA Profiling Reveals Unique miRNA Signatures in IGF-1 Treated Embryonic Striatal Stem Cell Fate Decisions in Striatal Neurogenesis *In Vitro*

**DOI:** 10.1155/2014/503162

**Published:** 2014-09-01

**Authors:** Soumya Pati, Nor Entan Supeno, Sangu Muthuraju, Raisah Abdul Hadi, Abdul Rahman Izaini Ghani, Fauziah Mohamad Idris, Mirjana Maletic-Savatic, Jafri Malin Abdullah, Hasnan Jaafar

**Affiliations:** ^1^Department of Neuroscience, School of Medical Sciences, Universiti Sains Malaysia, 16150 Kubang Kerian, Kelantan, Malaysia; ^2^Department of Pediatrics-Neurology, Baylor College of Medicine, Jan and Dan Duncan Neurological Research Institute at Texas Children's Hospital, 1250 Moursund Street, Room 1250, Houston, TX 77030, USA; ^3^Department of Pathology, School of Medical Sciences, Universiti Sains Malaysia, 16150 Kubang Kerian, Kelantan, Malaysia; ^4^Laboratory of Neuropsychopharmacology, FFCLRP, University of Sao Paulo (USP), Avenida Bandeirantes 3900, Monte Alegre, Ribeirao Preto, SP 14040-900, Brazil; ^5^Department of Medical Microbiology, School of Medical Sciences, Universiti Sains Malaysia, 16150 Kubang Kerian, Kelantan, Malaysia; ^6^Departments of Pediatrics-Neurology and Neuroscience, Program in Developmental Biology, Program in Structural and Computational Biology and Molecular Biophysics, Baylor College of Medicine, Jan and Dan Duncan Neurological Research Institute at Texas Children's Hospital, 1250 Moursund Street, Room 1250, Houston, TX 77030, USA; ^7^Center for Neuroscience Services and Research, Universiti Sains Malaysia, Sultanah Zainab 2 Road, 16150 Kubang Kerian, Kelantan, Malaysia; ^8^Neuroscience Department, Universiti Sains Malaysia Hospital, USM Hospital Road, 16150 Kubang Kerian, Kelantan, Malaysia

## Abstract

The striatum is considered to be the central processing unit of the basal ganglia in locomotor activity and cognitive function of the brain. IGF-1 could act as a control switch for the long-term proliferation and survival of EGF + bFGF-responsive cultured embryonic striatal stem cell (ESSC), while LIF imposes a negative impact on cell proliferation. The IGF-1-treated ESSCs also showed elevated hTERT expression with demonstration of self-renewal and trilineage commitment (astrocytes, oligodendrocytes, and neurons). In order to decipher the underlying regulatory microRNA (miRNA)s in IGF-1/LIF-treated ESSC-derived neurogenesis, we performed in-depth miRNA profiling at 12 days* in vitro *and analyzed the candidates using the Partek Genome Suite software. The annotated miRNA fingerprints delineated the differential expressions of miR-143, miR-433, and miR-503 specific to IGF-1 treatment. Similarly, the LIF-treated ESSCs demonstrated specific expression of miR-326, miR-181, and miR-22, as they were nonsignificant in IGF-treated ESSCs. To elucidate the possible downstream pathways, we performed* in silico *mapping of the said miRNAs into ingenuity pathway analysis. Our findings revealed the important mRNA targets of the miRNAs and suggested specific interactomes. The above studies introduced a new genre of miRNAs for ESSC-based neuroregenerative therapeutic applications.

## 1. Introduction

The striatum of the human brain participates in the control of the higher-level organisation of learning. It represents a crucial element in the neural circuitry of underlying procedural learning, motor control, reward-oriented learning, and the prediction of error signals [1]. Damage to the striatum could lead to persistent cognitive dysfunction [2]. A few studies that investigated the multipotential attributes of embryonic striatal stem cells (ESSCs) have demonstrated that these cells produce less dopaminergic neurons in the glomerular layer than cortex-derived neural precursor cells [3]. Similar to other neural stem cells, ESSCs change their properties during propagation* in vitro*, in tandem with the increasing number of passages, as shown by a decreased rate of proliferation [4]. Although ESSCs have shown the differentiated phenotypes of astrocytes and neurons under the influence of epithelial growth factor (EGF) and fibroblast growth factor (FGF) [5, 6], their long-term lineage plasticity* in vitro* is yet to be characterized.

Recent studies from our lab have demonstrated that insulin growth factor-1 (IGF-1) treatment controls long-term proliferation and the enhanced survival of epithelial growth factor + basic fibroblast growth factor- (EGF + bFGF-) responsive rat ESSCs, while leukemia inhibitory factor (LIF) negatively regulates the proliferation of these cells [7]. IGF-1 can act on both EGF and bFGF, and might modulate their actions during neurogenesis via the extracellular signal-related kinase (ERK)/mitogen-activated protein kinase (MAPK) pathway [8]. LIF treatment, on the other hand, has been found to attenuate the survival of cortical precursor cells from late rat embryos (beyond embryonic day 16, E16) by abrogating the generation of terminal lineages via the activation of the transcription factor STAT3 [9].

To further elucidate the molecular and cellular basis of IGF's role in the plasticity of ESSCs, we sought to investigate self-renewal, telomerase expression, and trilineage commitment (astrocytes, oligodendrocytes and neurons) in IGF-1-treated ESSCs and to determine the underlying microRNA (miRNA) regulatory pathways involved. Our findings have exposed the intrinsic miRNA signatures of the IGF-1 treatment of ESSCs. Finally, the miRNA-dependent downstream cascade analysis has unravelled the unique mRNA targets, and their primary mRNA interactomes responsible for ESSC fate specification. These miRNAs could be the next generation candidates for neuroregenerative cell therapies.

## 2. Materials and Methods

### 2.1. Isolation of Embryonic Rat Striatal Tissue

Time-mated Sprague-Dawley rats containing embryos at gestation day 18 were used for the isolation of striatal precursor cells from the striatum. The animal protocol was ethically approved by the Animal Research Unit, Universiti Sains Malaysia, Malaysia. The E18-derived striatal precursors were isolated and cultured according to previously published methods [7, 24] with a few modifications. Rat's embryos were dissected on E18. The pregnant Sprague-Dawley rats were sacrificed by deep anesthesia using anaesthesia cocktail consisting of ketamine (44 mg/kg) and xylazine (5.0 mg/kg). The rat's abdomen was shaved using a razor. After that, the shaved area was washed with 70% ethanol and wiped using sterile gauze. With sterile scissor and forceps, a lateral cut was made across the lower abdomen just anterior the vaginal orifice. The skin was retracted to the left and right side before a cut was made through the muscle layer. The uterine horns were then removed and placed immediately in sterile Petri dish on ice. The embryos at 18 days post-conception were then removed from their individual sacs and placed in sterile Phosphate Buffer Saline with 6% glucose (PBSg) on ice. The embryos were then immersed in 70% ethanol before being decapitated. All procedures were done in Class II biosafety cabinet. The striatal region of the embryo's brain was identified based on the morphology and also anatomically identified by its signature blotchy area using stereoscopic dissecting microscope. Intense care was taken to reduce the amount of connective tissue in the sample. The striatal tissues were then isolated and finally pooled into a 50 mL falcon tube containing 15 mL of PBSg containing 1% penicillin/streptomycin. For single-cell preparation, we used the Detachin Cell Detachment Solution (Genlantis, Gene Therapy Systems Inc., USA) immediately following dissection [25, 26]. Following the Detachin treatment, the cells were mechanically dissociated using tools with three different diameters (1 mL tip, 23 G syringe and 21 G syringe), and filtered through a 40 *μ*m cell strainer (BD Falcon). The cell density was determined by using a haemocytometer, and the trypan blue exclusion assay was used to determine the cell viability. In every operation, approximately 5 embryos generated up to 10 × 10^6^ cells.

### 2.2. Preparation of Media and Growth Factor Conditions

**Figure 1:**
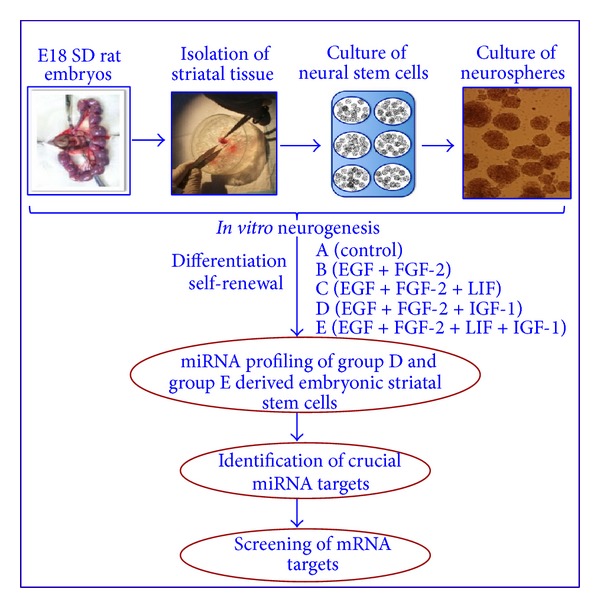
Strategy used for isolation of neural stem cells (NSCs) from E18 derived SD rat embryos and study of* in vitro* neurogenesis. The strategy of the experimental design shows four major steps that is, isolation of E18 embryos, enumeration of striatal tissue, culture of NSCs and culture of neurospheres. The schematic presentation also described the objectives and different growth factor combinations used for NSCs.

E18 ESSCs were cultured using the NeuroCult NS-A Proliferation Kit and NeuroCult NS-A Differentiation Kit (rat) from Stem Cell Technologies, for the induction of proliferation and differentiation, respectively. The growth factor supplements consisting of insulin-like growth factor-1 (IGF-1, Sigma I 8779), leukaemia inhibitory factor (LIF, Sigma L 5158), epidermal growth factor (EGF, Sigma E 4127) and basic fibroblast growth factor (bFGF, Sigma F 0291) were purchased from Sigma. The concentrations of these growth factors used were 20 ng/mL for the EGF and bFGF, 100 ng/mL for the IGF-1 and 20 ng/mL for the LIF, in accordance with our recently published report [7]. The ESSCs were plated in T-25 culture flasks (BD Falcon) in triplicate, under five different conditions (Figure 1): group A (no growth factor); group B (EGF + bFGF); group C (EGF + bFGF + LIF); group D (EGF + bFGF + IGF-1); and group E (EGF + bFGF + LIF + IGF-1).

### 2.3. Passaging of Embryonic Striatal Stem Cells

ESSC-derived neurospheres were passed into different cell culture flasks for* in vitro* expansion. The neurospheres were passed every fourth day* in vitro* (DIV); and single cell dissociations were done once the diameters of 150 to 200 *μ*m were reached. These neurospheres were then processed by two stage centrifugation, which was at 400 rpm for 5 minutes, followed by 1600 rpm for 6 minutes [25]. The supernatant was gently removed, and the cell pellets were then incubated with 1% Detachin for 10 minutes at 37°C. Following incubation, the neurospheres were triturated: first by using a Pasteur pipette, then by using a 1 mL pipette tip, and finally with a 23 G syringe needle, with 10 to 15 minutes for each step as previously described [26]. The cells were then seeded into a complete medium containing growth factors, following a final stage of centrifugation at 1600 rpm for 5 minutes.

### 2.4. Plating for Differentiation Experiments

At each cell passage and for each experimental condition, precursor cells were prepared as described above and plated onto 8-well chamber slides (BD Falcon), at a density of 50,000/50 *μ*L in a NeuroCult differentiation medium, with the proposed growth factor conditions (Figure 1). The cells were grown in a 5% CO_2_ incubator at 37°C for up to 5 passages. At each passage, the chamber slides were removed and stained for the presence of neurogenic markers, and the results were analysed with confocal microscopy (Zeiss).

### 2.5. Characterisation of E18-Derived Embryonic Striatal Stem Cells

The ESSCs were assayed for neural lineage commitment on different days using the Neural Stem Cell Marker Characterisation Kit (Chemicon, Millipore, USA), which included antibodies against nestin, Sox2, Map2, GFAP, and oligodendrocyte marker O1. All of the procedures were done in accordance with the manufacturer's instructions. The cell samples were further analysed by laser scanning microscopy (Pascal 5 confocal microscope, Carl Zeiss, Germany).

### 2.6. Telomerase Expression in ESSCs

The synergistic effect of the growth factors (Figure 1) on senescence was analysed in striatal progenitors and lineage-committed cells by quantifying the telomerase level at the 12th DIV. A rabbit polyclonal antibody against TERT (H-231) (sc-7212, Santa Cruz) was used in flow cytometry analysis. The antibody recognises an epitope corresponding to amino acids 900–1130 of telomerase reverse transcriptase (TERT). In total, 1 × 10^6^ cells per well were seeded in 96-well microplates. The cells were washed twice with PBS-containing 1% BSA, and later centrifuged at 1600 rpm at 4°C. The cell pellets obtained were fixed with chilled methanol, and then air dried, followed by washing three times with PBS-containing 1% BSA. The cells were then blocked with 10% normal blocking serum in PBS to avoid non-specific binding of the cells. HeLa cells were used as the positive control for telomerase activity. The primary antibody used was diluted 1 : 50 in 1.5% normal blocking serum. The cells were labelled with the primary antibody and maintained for 60 minutes at room temperature (RT), while the negative control was prepared without any primary antibody. The FITC-conjugated specific secondary antibody was diluted (1 : 100) in 1.5% normal blocking serum. The cells were then incubated for 45 min at RT in a dark chamber. Finally, the cells were washed three times and analysed by flow cytometry using a BD FACSCalibur.

### 2.7. Isolation of RNA and miRNA Hybridization and Data Acquisition

The RNA samples were isolated from triplicates of three experimental cell groups (treatments A, D, and E) (Figure 1), using the Qiagen miRNeasy Mini Kit. Briefly, the RNA was subjected to spectrophotometric measurements (BioSpec-mini, Shimadzu), and its quality was determined using an Agilent Bioanalyzer. Then, 1000 ng of the total RNA was Poly (A) tailed and ligated to a biotinylated signal molecule using the FlashTag Biotin HSA RNA Labelling Kit. The biotinylated target RNA samples were then hybridized to Affymetrix GeneChip miRNA arrays (Origen labs, 4110654) for 16 hours at 48°C, with rotations at 60 rpm.

This array provides probe sets for 71 species (human, mouse, rat, canine, monkey and more) comprising more than 46,000 unique probe sets that constitute over 6,703 miRNA sequences, with 922 Human snoRNA and scaRNA sequences. The probe set arrays were then washed and stained using the FS450_0003 fluidics protocol, and scanned using an Affymetrix 3000 7G scanner. The scanned images were inspected for hybridization efficiency, and the CEL files generated from the AGCC (Affymetrix GeneChip Command Console) were imported into the miRNA quality control QC Tool software for the determination of data quality.

### 2.8. MiRNA Data Processing Analysis

The MiRNA array data analysis was performed with the Partek Genomics Suite software. For the evaluation of the miRNA expression data, Affymetrix CEL files were imported into the software prior to the analysis. The miRNA data processing was performed as suggested by the Partek Genomics Suite. The hierarchical clustering analysis of the miRNA expression was performed using CLUSTER 3.0/Tree View software. The miRNA profile data was published by the Gene Expression Omnibus (GEO) database (GEO; http://www.ncbi.nlm.nih.gov/geo/) with the accession number* GSE30276*.

### 2.9. Statistical Analysis

The two-way ANOVA was done for the miRNA profiles using the Partek Genomics Suite based software. A Kruskal Wallis test was performed, followed by the Mann-Whitney-*U* test to analyse the data for telomerase expression. All data was expressed as the mean ± SD. A *P*-value < 0.05 was considered to be statistically significant in all experiments.

## 3. Results and Discussion

### 3.1. Effect of IGF-1 on Self-Renewal and Lineage Commitment by ESSCs

**Figure 2:**
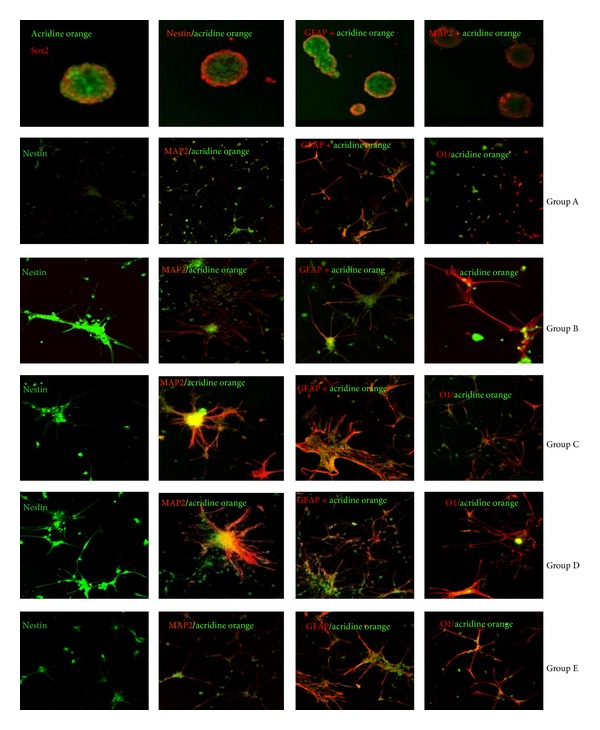
Lineage commitment and self-renewal by IGF-derived ESSCs at 20 DIV—Differentiated phenotypes of ESSCs were characterized on 20th DIV using several stage specific markers. Immunoflouroscence micrographs displayed appearance of self-renewing ESSCs by SOX-2 and nestin expressing neurospheres. Representative confocal images depict generation of oligodendrocytes, astrocytes and neurons as O1+ cells, GFAP+ cells, and MAP-2+ cells respectively in ESSCs derived from different treatment groups (A to E). Nuclei were stained by acridine orange counter staining. Bar = 100 *μ*m.

IGF-1 signalling controls a nexus of diverse molecular crosstalks involved in the proliferation and differentiation of embryonic and adult neural progenitors [27]. However, no supporting evidence existed for its effect on self-renewing activity and trilineage formation by ESSCs. Recent findings from our lab suggested that IGF-1 is important for the survival and long-term proliferation of EGF + bFGF-responsive ESSCs [7]. Using this existing knowledge of the IGF's multidimensional roles, we assumed that IGF-1 might also contribute to maintaining the striatal stem cell pool and trilineage commitment. To evaluate this hypothesis, ESSCs from E18 rat embryos were grown* in vitro* as neurospheres in five groups of experimental conditions, each having different combinations of growth factors, that is, group A, (without growth factor); group B, (EGF + bFGF); group C, (EGF + bFGF + LIF); group D, (EGF + bFGF + IGF-1); and group E, (EGF + bFGF + LIF + IGF-1) (Figure 1). The neurospheres showed mature morphology and the prominent expression of SOX2 and Nestin for the IGF-1 derived population at the 20th DIV, as demonstrated by immunofluorescence microscopy (Figure 2), whereas the LIF treatment alone could not result in such maturity and sustenance (data not shown). Interestingly, among all these groups of neurospheres, the clonal cells that were exposed to IGF-1 randomly differentiated into astrocytes, oligodendrocytes, and neuronal phenotypes, as represented by the expression of GFAP, O1, and MAP2 in these specific cells at the twentieth DIV, respectively (Group D, Figure 2). However, the (EGF + bFGF)-treated ESSCs hardly showed any differentiated phenotypes, and the LIF treatment alone did not show any mature oligodendrocytes at all (Group C, Figure 2). We then pondered whether the addition of IGF-1 to the LIF-treated ESSCs could restore the formation of oligodendrocytes, since IGF-1 had earlier been shown to direct the differentiation of adult neural precursors into oligodendrocytes [28]. Our results confirmed that the IGF-1 addition is absolutely crucial for ESSC-derived oligodendrocyte lineages. In summary, IGF-1 treatment is important for the maintenance of the ESSC pool, as well as trilineage formation.

### 3.2. Effect of IGF-1 on Telomerase Expression in ESSCs

**Figure 3:**
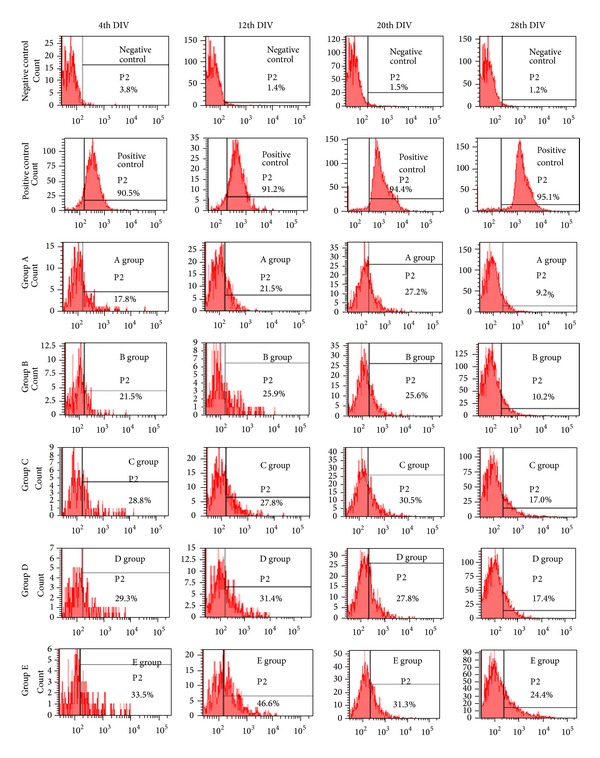
Telomerase expression of ESSCs—Expression of telomerase reverse transcriptase, TERT was evaluated in LIF + IGF-1 treated ESSCs to detect changes in inherent telomerase expression at fourth DIV, twelfth DIV, twentieth DIV, and twenty eighth DIV* in vitro*. Flow cytometry based analysis revealed differential expression of TERT in ESSCs from different groups.

**Figure 4:**
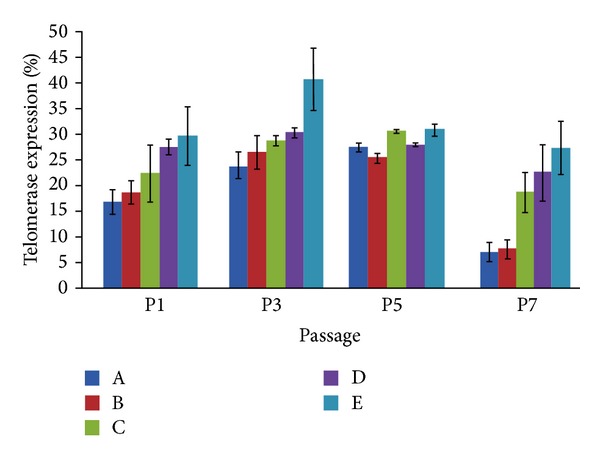
Telomerase expression of ESSCs. Telomerase expression of ESSCs at different time points. Kruskal Wallis test was performed, followed by Mann-Whitney-*U* test. *P* < 0.05 was considered significant. It revealed that combinatorial treatment of LIF along with IGF-1 (group E) showed maximum TERT expression at 12th DIV as compared to all time points.

Accumulating evidence has shown that telomerase expression is a regulatory checkpoint for priming stem cells for maintaining self-renewal and, ultimately, controls the epigenetic balance from aging to senescence [29]. It is also worth noting that the role of telomerase enzymes is strongly associated with defective neurogenesis and neuritogenesis [30]. Since an IGF-1 fortified cell culture would invariably support the cell fate discriminations in ESSCs, comprising the self-renewal-to-differentiation status, we hypothesized that IGF-1 might also regulate telomerase expression in ESSCs. To further explore the effects of IGF-1 on telomerase expression in (EGF + bFGF)-treated ESSCs, we elucidated the time dependent expression of the telomerase reverse transcriptase, TERT, at the fourth, twelfth, twentieth and twenty eighth DIVs by flow cytometry (Figures 3 and 4). Our findings suggest that IGF-1 treatment alone could elevate the TERT levels, but a combined treatment with LIF significantly enhanced TERT expression at the 12th DIV, as compared to other time points (Figures 3 and 4). This upregulation of the TERT expression in the ESSCs treated with either IGF-1 alone or in combination with LIF strongly represented two important possibilities: IGF-1 regulates the TERT expression in ESSCs, and LIF might also be playing a direct or indirect role in modulating the TERT levels to maintain the self-renewal of ESSCs to restore a stable ESSC pool. These findings directly coincide with some earlier published reports, which demonstrated that IGF upregulates the human telomerase reverse transcriptase (TERT) by Akt-based phosphorylation and that the telomerase reactivation could reverse neurodegeneration with the restoration of proliferating SOX2+ neural progenitors and Olig1+ oligodendrocyte populations in telomerase-deficient mice [31, 32]. Our findings also support this, since the ESSCs from the IGF-treatment group restored both the SOX2+ and O1+ cells at the twentieth DIV (Figure 2(C)), wherein the LIF treatment controlled the number of neural precursors by abrogating the emergence of more differentiated cell types. This occurred via the activation of the transcription factor STAT3 and could not help the survival of the cortical precursor cells isolated from later gestation rat embryos (beyond embryonic day 16, E16) [33]. Based on the above information, our findings confirmed that IGF-1 in combination with LIF might play a key role in senescence by modulating the TERT levels during early stages of ESSC-derived neurogenesis.

### 3.3. MicroRNA Microarray of Cultured ESSCs

MicroRNA is one of the key epigenetic factors involved in cellular senescence. Although several studies have exposed the role of specific microRNAs in the aging of neural stem cells, there is hardly any information available for miRNAs involved in striatal stem cells. Based on a recent finding from our lab, involving IGF-1's role in the long-term proliferation and survival of ESSCs [7], as well as our present data; we hypothesised that IGF-1's multifunctional attributes in ESSCs may require the recruitment of crucial miRNAs and their unique downstream pathways. To further evaluate the cellular and molecular changes introduced in ESSCs by LIF and IGF-1, and to delineate the specific miRNAs involved, we have performed in-depth miRNA profiling in ESSCs (group D and group E, Figure 1) at the 12th DIV. We analysed the miRNA profile using the Partek Genome Suite (PGS) software. The 12th DIV specific time point was selected on the basis of our recent report, which had shown the maximum increment in proliferation in IGF-1-treated ESSCs during that time point [7]. This was also based on our present data, which represented a sound telomerase expression at the same time point. We would like to emphasize that since the LIF treatment alone was detrimental for the long-term proliferation and survival of ESSCs [7] and IGF-1 could give a surge to the telomerase expression in the LIF-treated group (Figure 3), we decided to perform miRNA profiling in IGF-1 alone and the LIF/IGF-1 treated ESSC populations.

**Figure 5:**
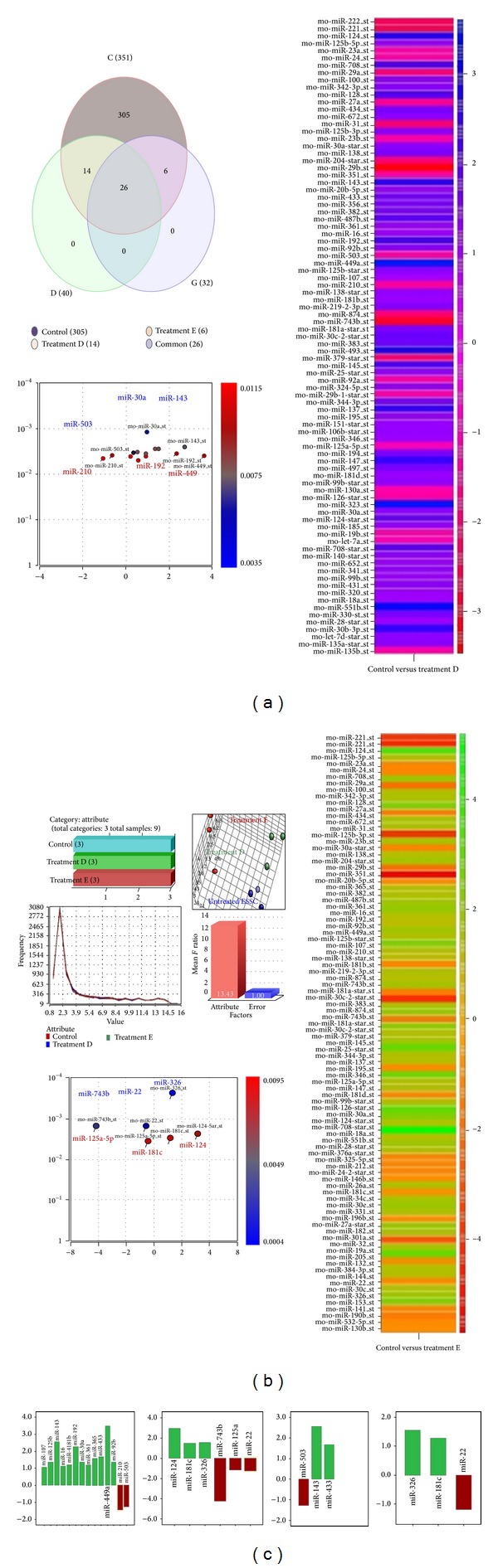
(a) A Venn diagram displayed the common and differentially expressed miRNAs in control and treated striatal stem cells. The profiling had revealed a distinct set of 26 common candidates among both group D and group E derived ESSCs. (b) Partek Genomics Suite based analysis of miRNA profiles. The genome analysis software imports affymetrix-CEL files for each data set.*Assignments of sample attributes or the Categorical attributes*:These attributes are imposed to the imported data sets (*n* = 3) as per their specification such as treated, untreated, and nature. Assignment of sample attributes was displayed in three different rows.* PCA analysis for sample variation*: Principal components 1, 2, and 3 in 3D space represented approximately 49.7% of the variation in the scatter plot. Three main clusters were observed among the differentiating cells, including: (1) Embryonic striatal stem cells, ESSCs without any growth factor (red), (2) ESSCs from treatment group D (blue), and (3) ESSCs from treatment group E (green).* Validation of sample clusters. *The histogram represents the visualization of data distribution or the variation in the data analysis by the Partek software. Three samples showed similar frequency, revealing no discrepancy among these three samples, prior to sample analysis.* Sources of variation between samples*: Sources of variation or error were predicted for data using all test variables in the ANOVA model. The variation bar chart showed “signal-to-noise ratio” or “*F *ratio” in the* y*-axis. The “mean* F*-ratio” was the mean signal to noise ratios for all computed variables for the factors in* x*-axis.* Spatial patterning of miRNA distribution*: The representative volcano plots displayed spatial patterning of miRNA distribution for different groups on the basis of their* P* values and fold regulation. Each volcano plot is also shown along with corresponding heat maps indicating the list of candidates detected in both control versus treatment groups. (c) Fourteen differentially expressed miRNAs were detected in group D striatal stem cells. Out of these, twelve were found to be upregulated and two were downregulated. For the group E, six differentially expressed miRNAs were detected where three of the miRNAs were found to be up regulated while another three were down regulated. Among all these miRNAs, ESSC-specific candidates were selected on the basis of significant differential expression. IGF-1-derived ESSCs (group D) demonstrated significant upregulation of miR-143, miR-433 and downregulation of miR-503. ESSCs from group E (LIF/IGF-1 combinatorial effect) displayed upregulation of miR-181, miR-326 and downregulation of miR-22.

The samples were validated using data distribution and the sources of the variation parameters in the PGS (Figure 5(b)). These data were further authenticated for integrity and sample uniformity. Our samples maintained similar run frequencies with no discrepancy, and had a low signal-to-noise ratio (Figure 5(b)). The miRNA profiles of these IGF-1 and LIF + IGF-1 treated ESSCs were already accepted and published by the GEO databank of the NCBI (*GEO *accession number* GSE30276*), and the miRNA profiling resulted in 351 miRNA candidates in ESSCs at the 12th DIV. Based on the high throughput analysis by PGSE, we identified 26 common miRNAs among the above two cell groups, and 14 and 6 differentially expressed miRNAs between the IGF-1 and (LIF + IGF-1) treated ESSCs, respectively (Figures 5(a) and 5(c)). Among these miRNAs, with a stringency filter of −1 to +1-fold, we finally detected miR-503, miR-433, and miR-143 as being significant and differentially expressed (*P* < 0.01) in the IGF-1-treated ESSCs (Figure 5(c) and Table 1). The miR-326, miR-22, and miR-181c were significantly and specifically expressed in the (LIF + IGF-1)-treated ESSCs (*P* < 0.01) (Figure 5(c) and Table 1). These miRNA signatures suggest a set of distinctive regulatory cascades important for IGF-1 and LIF mediated signalling in ESSCs.

### 3.4. MiRNA Signatures in IGF-1-Treated ESSCs

#### 3.4.1. Identification of mRNA Targets and Ingenuity Pathway Analysis

The analyses of the miRNA profiles led us to further identify their direct mRNA interactomes and the downstream pathways involved in the ESSC fate specifications. To investigate the best possible downstream cascades regulated by these miRNAs and their primary interactomes, we used a licensed version of the ingenuity pathway analysis (IPA) software, which creates molecular networks of interactions with uploaded genes or miRNA candidates. To build a pathway and to detect the closest interactome, the IPA utilizes several databases involving direct or indirect gene relationships. The in-depth analysis of the pathways has deciphered the complex interplay of mRNA targets and suggested their possible roles in ESSC fate determination* in vitro*. All the detected primary mRNA targets were listed in Table 1.

#### 3.4.2. Downregulated miR-503

**Figure 6:**
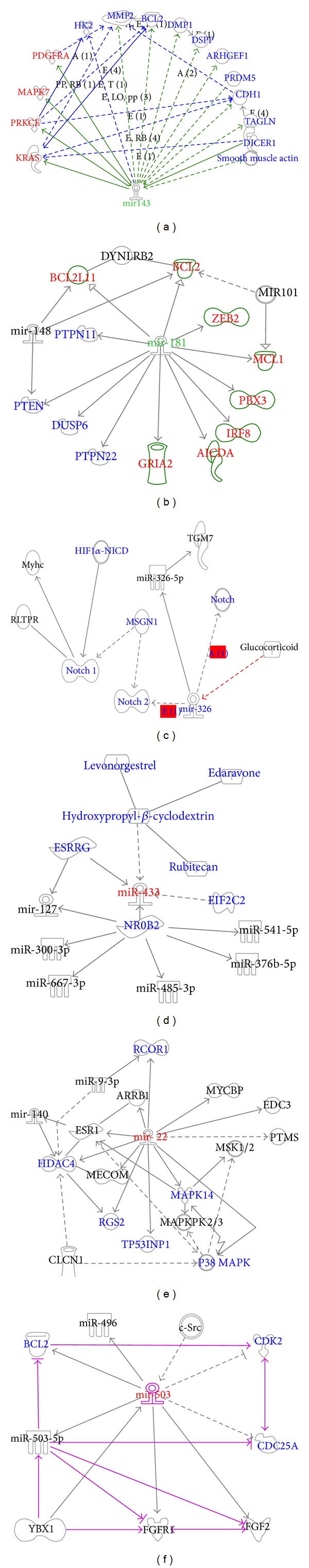
(a)–(f) Ingenuity pathway analysis based detection of downstream cascades of candidate miRNAs. Pathway detection and target identificationbased readout generated three categories of information including direct or indirect mRNA targets those get regulated, molecules that regulate miRNAs by physical interaction and possible primary interactomes of mIRNAs. The results have exposed potential mRNA targets of ESSC-specific miRNAs involved in their cell fate decisions.

The miRNA profile screening identified miR-503 as being significantly downregulated (1.35 times, *P* = 0.003) (Figure 6(a) and Table 1) in IGF-1-treated ESSCs. The downstream target analysis by the IPA revealed that miR-503 inhibits cyclin-dependent-kinase 2 (cdk2) by downregulating Cdc25A phosphatase and releases the inhibitory phosphorylation of cdk2, which was demonstrated earlier in the differentiating of myoblasts into myotubes and nonmuscle cells [10]. Since our findings suggested the downregulation of miR-503 in IGF-1-derived ESSCs, it is likely that the inhibition of cdk2 may be removed in the above population. Thus, we assume that the down-regulation of miR-503 might have induced the transition of the quiescence-to-early proliferative state in the striatal precursors under the influence of IGF-1, as IGF-1 had already been shown to enhance the early proliferation of EGF/bFGF-responsive ESSCs, compared to LIF, in a recently published report [7].

#### 3.4.3. Upregulated miR-433

The miRNA profiling of IGF-1-treated ESSCs has also revealed the significant upregulation of miR-433 (1.68 times, *P* = 0.002) (Figure 6(b) and Table 1). Further pathway analysis for miR-433 specific downstream copartners has revealed a few targets, such as oestrogen-related receptor gamma (ERRG), the nuclear receptor sub-family (NR0B2 or SHP-1), RNA-induced silencing complex (RISC) component EIF2, Argonaute 2 (Ago2), hydroxypropyl-beta-cyclodextrin, and others (Figure 6(b)). Some recent studies involving small RNA interfaces in the mouse brain have shown that both Ago2 and EIF2 exhibit specific roles in neurodevelopmental processes [11, 12], since EIF2C is specifically expressed in principal neurons, and the Ago2 knockout mice exhibited severe defects in neural development.

It is noteworthy that ERRG could enforce the simultaneous activation of miR-433 and miR-127, as both miRNAs are transrepressed by SHP-1. Interestingly, among the other targets of miR-433, we found two interesting progestogens (edaravone and hydroxypropyl-beta-cyclodextrin) that play important roles in neurodegeneration and neuroinflammation. Edaravone treatment substantially increased the proliferation of nestin+ neural stem cells, and also enhanced their numbers both* in vitro* and* in vivo*, whereas hydroxypropyl-beta-cyclodextrin (an ERRG agonist) could substantially increase miR-433 expression [13]. Based on our findings and pathway analysis, we hypothesize that elevated levels of miR-433 might be a signature of the fate switching of striatal precursors towards proliferation and lineage commitment under the influence of IGF-1.

#### 3.4.4. Upregulated miR-143

MiR-143 was upregulated 2.56 times (*P* = 0.002) in ESSCs under the influence of IGF-1 (Figure 6(c) and Table 1). The downstream cascade analysis of miR-143 exposed many multidimensional targets, such as platelet derived growth factor receptor alpha (PDGFRA), protein kinase C epsilon (PRKCE), and mitogen activated protein kinase 7 (MAPK7) (Figure 6(c) and Table 1). In addition to these targets, we also found two interesting matrix mineralizing proteins, dentin sialophosphoprotein (DSPP) and dentin matrix acidic phosphoprotein (DMP1) that also play roles in the internalization and localization of macromolecules present in the extracellular matrix (Figure 6(c) and Table 1). Further analysis of the interactomes suggested that the phosphorylated intracellular domains of PDGFRA play significant roles in the commitment of oligodendrocytes, astrocytes, and neuroprogenitors [14]. It has already been established that MAPK7 regulates proliferation, neuronal differentiation, and survival during embryonic development and neuronal differentiation in the adult subgranular zone, SGZ [15]. This pathway analysis also demonstrated KRAS and BCL-2 as the two direct targets of miR-143 that get downregulated. Together with these findings, it is strongly evident that IGF-1 treatment may be regulating the above targets, thereby substantially inducing the enhanced proliferation, survival, and differentiation of ESSCs* in vitro*.

### 3.5. MiRNA Candidates Detected in LIF-Treated ESSCs

#### 3.5.1. Upregulated miR-326

The miRNA profiles of LIF-treated ESSCs demonstrated a significant upregulation of miR-326 (1.58 times, *P* = 0.0002)* in vitro* (Figure 6(d) and Table 1). Further target identification and pathway prediction by IPA have revealed two interesting facts (Figure 6(d)). The mature miR-326 regulates transglutaminase 7 (TGM7), which is an essential enzyme involved in the catalysis of the tubulin polyamination that stabilizes the neuronal microarchitecture. Therefore, miR-326 might be involved in regulating the neuronal fate discrimination in LIF-instructed EGF + FGF-responsive striatal precursors [16]. Secondly, the Notch family members have been identified as the major interacting targets of miR-326 and established regulators of the number and survival of neural stem cells, both* in vitro* and* in vivo* [17]. This data strongly supports our previous findings, which suggested that LIF recruitment in cell culture might be attenuating the terminal cell differentiation in order to maintain the pool of neural stem cells. This is demonstrated as enhanced cell death or less metabolic viability in the long-term proliferation assay for ESSCs [7]. Therefore, it is assumed that the upregulation of miR-326 might be regulating the Notch signalling for maintaining the striatal precursor pool* in vitro*.

#### 3.5.2. Upregulated miR-181c

Our data further showed the significant upregulation of miR-181c at the 12th DIV (1.50 times, *P* = 0.0029) in LIF + IGF-1 treated ESSCs (Figure 6(e) and Table 1). Target scanning revealed that, from among the top downstream targets, there were four major protein tyrosine phosphatases present, including protein tyrosine phosphatase nonreceptor type 11 (PTPN11), protein tyrosine phosphatase nonreceptor type 22 (PTPN22), phosphatase and tensin homolog (PTEN), and dual specificity phosphatase 6 (Dusp6) (Figure 6(e)). The loss of PTEN activity is associated with the enhancement of the neural stem cell pool's self-renewal [18]. The mutant Dusp6 allele, showing dominant postnatal lethality, hearing loss, and other phenotypes, is attributed to the inappropriate activation of FGFR signalling, a negative feedback regulator of FGFR signalling* in vivo* [19]. This negative regulation can impair PTPN11 activity and lead to the altered PDGFRA signalling involved during the commitment of oligodendrocytes, astrocytes, and neuroprogenitors [20]. All of these indicate that the disruptions of the above phosphatases by miR181c can push the cell fate discrimination to an off-balanced state, leading to an enhanced self-renewal of the ESSC pool, by abrogating the lineage commitment under LIF treatment.

In addition to the above mentioned targets, we also found three unique transcription regulators: pre-B-cell leukaemia homeobox 3 (PBX3), zinc finger E-box binding homeobox 2 (ZEB2), and interferon regulatory factor 8 (IRF8) (Figure 6(e)), of which PBX3 is involved in the retinoic acid derived neuronal commitment of mouse embryonal carcinoma P19 cells. Additionally, ZEB2 is critical for the regulation of telomerase expression and the early differentiation of mouse embryonic stem cells [21]. These results strongly coincide with our findings involving the LIF-treated striatal precursors, which showed a significant enhancement in telomerase expression when compared to other groups in the presence of IGF-1 (Figures 3 and 4). Therefore, we hypothesize that ZEB2 inhibition by miR-181c in LIF-treated ESSCs might be one of the possible mechanisms for self-renewal and elevated TERT expression* in vitro*.

### 3.6. Downregulated miR-22

The scanning of miRNA profiles from LIF + IGF-1 treated ESSCs also detected that miR-22 was significantly downregulated (1.22 times, *P* = 0.002) (Figure 6(f) and Table 1). To further elucidate the miR-22 specific downstream effectors involved in neurogenesis, we delved into the top 10 indirect and direct interactomes suggested by ingenuity based repositories (Figure 6(f)). Recent studies focusing on miR22 have suggested its role as a neuroprotective candidate in Huntington's disease (HD) models* in vitro* [22], as its overexpression regulates several mRNA candidates associated with abnormalities of HD pathology, such as histone deacetylase 4 (HDAC4), REST corepressor 1 (Rcor1), and the regulator of G-protein signalling 2 (Rgs2) [23]. The underlying phenomenon behind such attributes does include the inhibition of caspase activation, along with the regulation of the proapoptotic activities of mitogen-activated protein kinase 14/p38 (MAPK14/p38) and tumour protein p53-inducible nuclear protein 1 (Tp53inp1). The IPA analysis showed all of the above targets to be primary interactomes of miR-22 (Figure 6(f) and Table 1); thus, the downregulation of miR-22 in LIF + IGF-1 treated ESSCs might be the rationale underlying the diminished proliferation and less metabolic viability, as compared to IGF-1 alone [7]. This is suggested by its ability to induce the enhanced activities of caspase, MAPK14/p38, and Tp53inp1 in increasing cell death under LIF treatment. These findings suggest that LIF enforcement might be involved in the miR-22 mediated modulation of proliferation and apoptosis in ESSCs.

## 4. Conclusion

*In vitro* studies from our lab have suggested that IGF-1 acts as multidimensional rheostat in telomerase activity, self-renewal, and trilineage commitment of ESSCs. In addition, in-depth analysis of miRNA profiles and their* in silico* mapping to IPA-based data repositories have revealed two very important pieces information: (1) involvement of unique group of miRNAs specific to IGF treatment in ESSCs-derived neurogenesis and (2) prediction of the primary neighborhood molecules in the interaction. These compelling data involving crucial regulatory miRNAs in ESSCs-derived neurogenesis can be used as future therapeutic candidates for* in vitro* manipulation of ESSCs for neuroregenerative therapies.

## Figures and Tables

**Table 1:** 

miRNAs	*P* value	Up-/downregulation	Predicted function in neurogenesis	mRNA targets	Functional significance
miRNA503	0.003	1.35 times	transition of quiescence-to-proliferation stage	Cdk2/Cdc25A	[10]

miRNA433	0.002	1.68 times	Neural development proliferation/self-renewal-to-differentiation	ERRG, NR0B2 or SHP-1, RISC, EIF2, Ago2, edaravone, and hydroxypropyl-beta-cyclodextrin	[11–13]

miRNA143	0.002	2.56 times	Proliferation, neural differentiation, and survival	PDGFRA, PRKCE, MAPK7, DSSP, DMP-1, KRAS, and BCL-2	[14, 15]

miRNA326	0.0002	1.58 times	Maintenance and survival of striatal precursor pool	TGM7	[16, 17]

miRNA181c	0.0029	1.50 times	Switch for lineage-to-self-renewal and telomerase expression	PTPN11, PTPN22, DUSP6, PBX3, IRF8, and ZEB2	[18–21]

miRNA22	0.001	1.22 times	Proliferation and apoptosis	HDAC4, RCOR1, RGS2, MAPK14/p38, Tp53inp1, and P38 MAPK	[22, 23]
